# Interferon-stimulated Gene 15 (ISG15) is a trigger for tumorigenesis and metastasis of hepatocellular carcinoma

**DOI:** 10.18632/oncotarget.2316

**Published:** 2014-08-06

**Authors:** Chong Li, Ji Wang, Hong Zhang, Mingao Zhu, Feifei Chen, Yufeng Hu, Hudan Liu, Hong Zhu

**Affiliations:** ^1^ Department of Oncology, the First Affiliated Hospital of Soochow University, Suzhou, China; ^2^ CAS Key Laboratory of Infection and Immunity, Institute of Biophysics, Chinese Academy of Sciences (CAS), Beijing, China; ^3^ Department of Oncology, the Second Affiliated Hospital of Soochow University, Suzhou, China; ^4^ School of Pharmacy, Tongji Medical College, Huazhong University of Science and Technology, Wuhan, China

**Keywords:** ISG15, HCC, Metastasis, Tumorigenesis

## Abstract

Hepatocellular carcinoma (HCC) is one of the most common cancers worldwide with poor prognosis. IFN-stimulated genes 15 (ISG15) is an ubiquitin-like molecule that is strongly upregulated by type I interferons as a primary response to diverse microbial and cellular stress stimuli. However, the role of ISG15 in HCC remains unclear. In this study, we investigated the function of ISG15 during HCC progression and related mechanism using clinicopathological data, cell line and xenograft model. Our results indicated that ISG15 is highly expressed in HCC tissues and multiple HCC cell lines. ISG15 expression is significantly associated with the differentiation grade, metastatic of tumor and survival of HCC patients. However, the expression of ISG15 is not affected by HBV infection. ISG15 promotes the proliferation and migration of hepatocarcinoma cells through maintaining Survivin protein stabilization via sequestering XIAP from interacting with Survivin. Knowing down ISG15 with SiRNA inhibited the xenografted tumor growth and prolonged the lifespan of tumor-bearing mice. All these results support that ISG15 high expression is an intrinsic feature for HCC and a trigger for tumorigenesis and metastasis. ISG15 may be a prognostic biomarker and the inhibition of ISG15 could provide a therapeutic advantage for HCC patients over-expressing ISG15.

## INTRODUCTION

Hepatocellular carcinoma (HCC) is the sixth most common solid neoplasm and the leading cause of cancer-related death in the world with 55% occurring in China [1-3]. According to a recent report from the International Agency for Research on Cancer, HCC has become the second most common cause of cancer-related death and accounts for 9.1% (0.8 million) of the global cancer-related deaths in 2012 [4]. Despite advance in the treatment of HCC, there is currently no curative option for this life-threatening disease and the overall 5-year survival is about 40% for patients treated with major hepatectomy [5]. Although not satisfactory, molecularly targeted drugs, e.g. tyrosine kinase inhibitor sorafenib, have brought promising outcomes to HCC, which encourages the scientists to shift their interests towards the development of novel biotherapeutic agents. Furthermore, advances in molecular target are likely to derive from a better recognition and understanding of the biological behavior and pathogenesis. Chronic hepatitis B virus (HBV) infection accounts for 52% of the causes of HCC, followed by chronic infection with hepatitis C virus and alcoholic liver disease [6]. HBV is a well-known predominant etiologic risk factor and its carriers have a 100-fold relative risk for developing HCC with an annual incidence rate of 2–6% in cirrhotic patients [7]. Sustained inflammation caused by chronic HBV infection is not only involved in hepatocarcinogenesis, but also plays critical roles in the recurrence and metastasis of HCC after surgical treatment [8].

To survive from viral infection, cells can produce and secrete interferons (IFNs), proinflammatory cytokines which can block viral infection and replication, cellular proliferation, and inhibit important immunomodulatory activities. One important mechanism by which IFNs mediate their antiviral effects is through the transcriptional regulation of relevant genes, such as IFN-stimulated genes (ISGs) [9, 10]. Among them, ISG15 is an ubiquitin-like protein that conjugates to cellular substrates to form ISGylated proteins and shows antivirus activities [11]. The expression of ISG15 also can be regulated by IFN regulated factor (IRFs) and it was one of the first reported targets of IRF3, a 55 kDa protein that is constitutively expressed in all tissues. Viral infection induces phosphorylation and activation of IRF. Phosphorylated IRF3 translocates into the nucleus and activates the expression of ISG15 by binding to the ISRE/IRFE elements [12-15]. The structure of ISG15 and ubiquitin are highly homologous with the similar region, known as ubiquitin cross-reactive protein (UCRP) [16]. ISG15 is covalently conjugated with cellular proteins in an enzymatic pathway comprised of the activating E1, conjugating E2 and ligating E3 enzymes, which is similar to ubiquitylation. The conjugation of ISG15 with protein substrates provides a tag that either marks the labeled protein for degradation or modulates its function [16-18].

Mounting studies have identified specific alterations of ISG15 pathway in human tumors, such as bladder cancer, prostate cancer, breast cancer, colorectal cancer, and acute multiple sclerosis lesions [12, 19-23]. Like other innate immune/stress response mediators, appropriately regulated ISG15 expression is associated with a tumor suppressor function, whereas the perturbation of ISG15 regulation is correlated with enhanced tumor progression and leads to aberrant cell signaling and malignant transformation [12, 21, 24, 25]. Unlike ubiquitin whose expression is more or less constant in all cells, the ISG15 protein is highly expressed in the majority of tumor cells. Moreover, the expression of ISG15 is with a high degree of heterogeneity in both tumor cell lines and tumor tissues [26, 27].

Up to now, the role of ISG15 in HCC is still unclear and whether HBV infection-based HCC is correlated to the alterations of ISG15 expression remains to be determined. Here we explore the function of ISG15 in HCC progression and its mechanism using clinical pathological data, cell line and xenograft model. Our results disclose that high expression of ISG15 is an intrinsic feature for HCC and a trigger for tumorigenesis and metastasis. As a poor prognosis marker, the inhibition of ISG15 could provide a therapeutic advantage for HCC patients over-expressing.

## RESULTS

### ISG15 is highly expressed in HCC cell lines and cancer specimen

**Figure 1:**
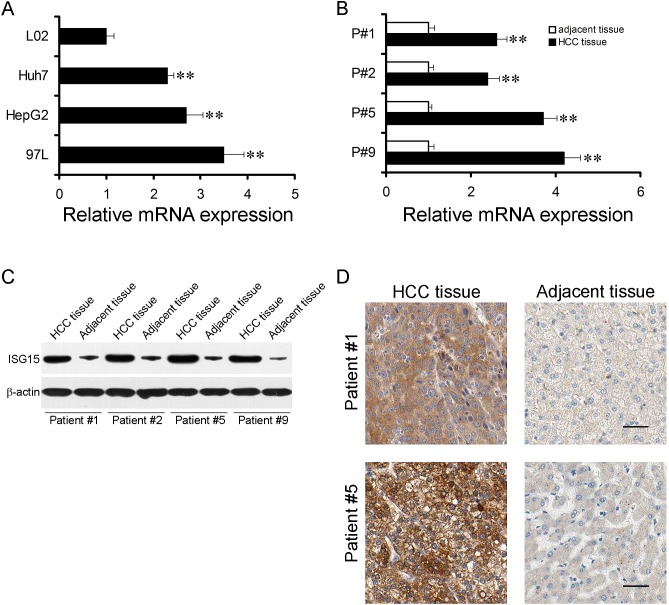
ISG15 is highly expressed in HCC cells and tissues The mRNA expression levels of ISG15 in HCC cells (A) and tissues (B). (A), Data were means from three independent experiments with three dishes for each cell line, bars, SD. (**, *P* < 0.01). (B), four representative results were shown (**, *P* < 0.01). The difference in Protein expression levels of ISG15 in HCC tissues was compared with the corresponding adjacent tissues by western blot and immunohistochemistry in 50 pairs of human HCC specimens. The representative results were shown in C and D.

We checked ISG15 mRNA level in HCC cell lines, Huh7, hepG2 and 97L with a non-HCC cell line L02 as controls. Real-time PCR revealed that ISG15 mRNA in HCC cell lines were higher than that in L02 (Figure 1A, *P* < 0.01). Next, we determined whether ISG15 overexpressed in HCC specimens compared to non-tumor counterparts. ISG15 mRNA level of fifty pairs of human HCC samples and their non-tumor counterparts were analyzed, which was 2.4 to 4.2 folds higher in HCC specimens (Figure 1B, *P* < 0.01). ISG15 protein levels were also examined in the HCC specimens (Figure 1C, D), among which 84% (42/50) of the cases showed relatively higher ISG15 expression than in the non-tumor counterparts (0.88 ± 0.07 vs. 0.50 ± 0.04, *P* < 0.001). Our data suggest that ISG15 level is higher in HCC.

### Expression of ISG15 is related to HCC histologic differentiation, metastasis and predicts worse 5-year survival

50 human HCC specimens were evaluated for the correlation between ISG15 protein levels and clinicopathologic features by univariate analysis, including patient's age, gender, HBV infection, alpha fetoprotein (AFP) level, number and size of the lesions, portal vein tumor thrombus and metastasis (Table 1). The results showed that ISG15 protein level was not affected by the patient's age, gender, HBV infection, AFP level, number and size of the lesions and portal vein tumor thrombus (*P* > 0.05). In contrast, the ISG15 protein levels were associated with poor HCC histologic differentiation and metastasis (*P* < 0.01).

**Table 1:** Relationship of the Expression of ISG15 and Clinicopathological Feature of HCC

Parameter	Groups	Number of Patient	ISG15 Relative Expression	*P*
Age(yr)	≥50	19	0.92±0.14	0.764
	<50	31	0.84±0.09	
Gender	Male	44	0.85±0.08	0.456
	Female	6	0.86±0.22	
HBV infection	Postive	46	0.89±0.08	0.555
	Negative	4	1.07±0.38	
AFP(ng/ml)	≥400	27	0.87±0.11	0.394
	<400	23	0.93±0.12	
Tumor size(cm)	≥5	29	0.88±0.12	0.680
	<5	21	0.84±0.09	
Tumor number	Single	39	0.86±0.09	0.210
	Multiple	11	1.22±0.13	
Differentiation	Moderate to well	33	0.54±0.06	<0.001^*^
	Poor	17	1.34±0.20	
Portal vein tumor thrombus	Positive	3	1.01±0.40	0.685
	Negative	47	0.86±0.08	
Metastasis	Positive	22	1.12±0.10	0.001^*^
	Negative	28	0.68±0.11	

**Figure 2:**
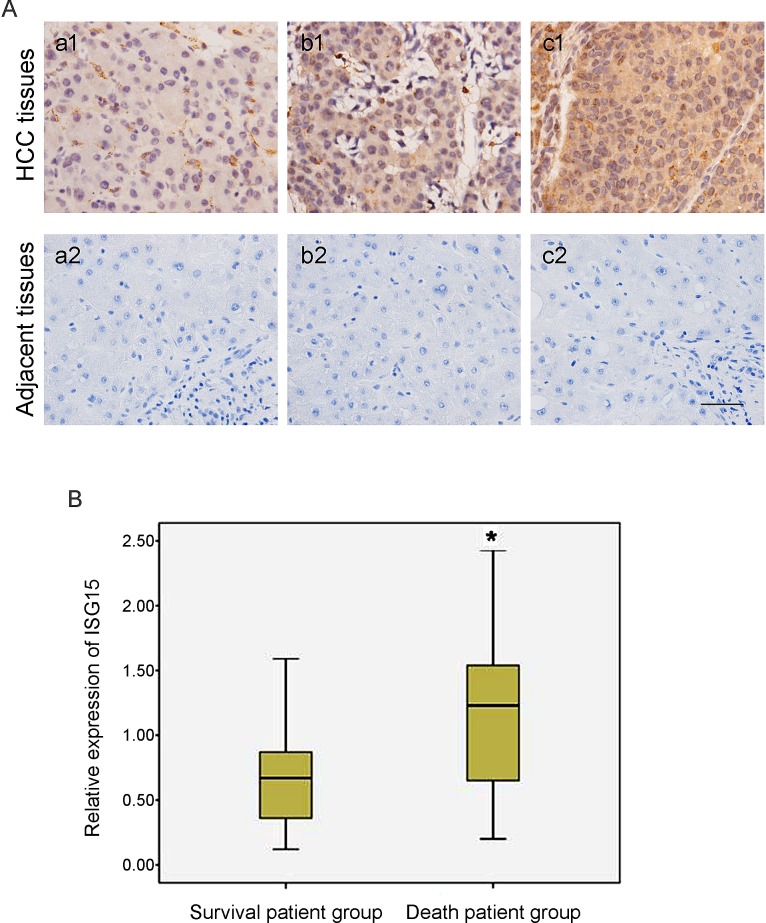
Expression of ISG15 is related to HCC histologic differentiation and the worse 5-year survival (A)HCC tumor tissues with histologic grade were examined to the expression of ISG15 by immunohistochemical analysis. The represented results were shown in (a-b) moderate to well HCC tissues and (c) poor differentiation HCC tissues. A strong immunochemical signal for ISG15 was detected predominantly in the cytoplasm. ISG15 expression was remarkably higher in poorly differentiated HCC tissues with respect to moderate to well differentiated HCC tissues, indicating that the expression of ISG15 protein was relevant to HCC differentiation status and malignancy grade. The black short line at bottom right indicates 50 μm. (B) The 50 HCC patients were divided into two groups according to patient's survival status at 5 year after being diagnosed pathologically as HCC. We found the expression of ISG15 was higher in the death patient group than in the survival patient group (Figure 2B, *P* = 0.034), suggesting that the high level of ISG15 increased the risk of death in HCC patients. The relationship between the expression of ISG15 and 5-year survival of HCC patients was analyzed by Pearson Chi-squared test. The height of box represented the interquartile range of ISG15 expression level and the black line in the middle of box represented the median. The short black line on top and bottom outside the box represented respectively the maximum and minimum of ISG15 expression amount in the involved HCC patients.

Immunohistochemical analysis confirmed simultaneously that ISG15 protein level was remarkably higher in poorly differentiated HCC tissues compared to moderate to well differentiated HCC tissues, suggesting that ISG15 was relevant to HCC differentiation status and malignancy grade (Figure 2A). Furthermore, considering that the protein level of ISG15 was related to HCC histologic differentiation and metastasis, we analyzed the relationship between the expression of ISG15 and 5-year survival of HCC patients by Pearson chi-square test. The HCC patients were divided into the survival patient group and death patient group according to patient's survival status at 5 year after being diagnosed pathologically as HCC. We found the expression of ISG15 was higher in the non-survivors at 5 years (Figure 2B, *P* = 0.034), suggesting that ISG15 is a prognostic marker for worse 5-year survival.

### Knocking down ISG15 inhibits cancerous proliferation, migration and arrested cell cycle at G2/M phase

**Figure 3:**
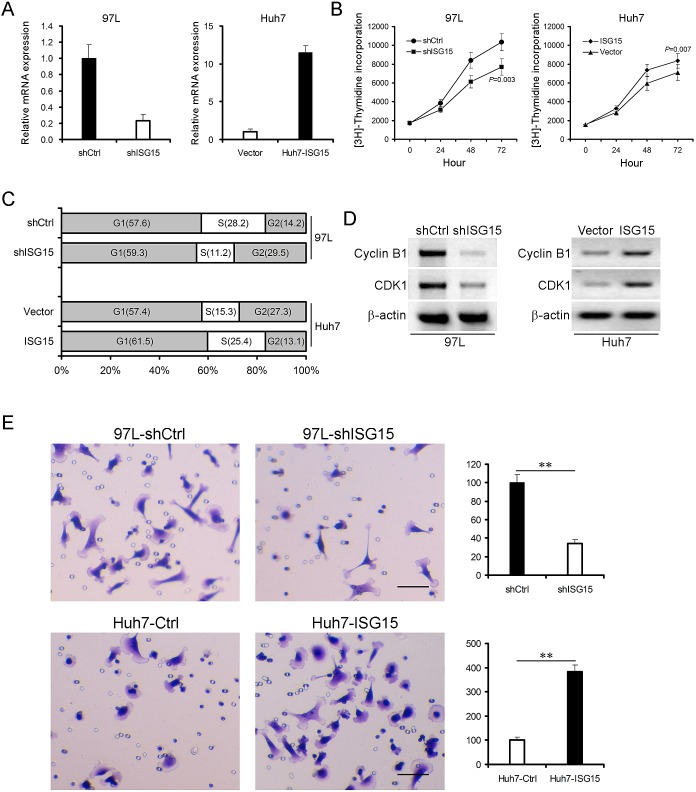
ISG15 promotes cancerous proliferation, migration and involves in cell cycle ISG15 was knocked down in 97L cells (97L-shISG15) (A, left pannel) and was overexpressed in Huh7 cells (Huh7-ISG15) (A, right panel). β-actin was probed as a control. (B) 97L-shISG15 cells (left panel, P = 0.003) or Huh7-ISG15 cells (right panel, *P* = 0.007) were cultured from 24 to 72h followed by incubation with [^3^H]-thymidine for 4 h. Data are from three independent experiments with three dishes and shown as means ± SD. (C) 97L-shISG15 cells or Huh7-ISG15 cells were analyzed to detect respectively their cell cycle by flow cytometry using propidium iodide (PI) staining. (D) Cell lysates were immunoblotted with antibodies against Cyclin B1 or CDK1. β-actin was used as a loading control. (E) Trans-well migration assays showed that there was a positive correlation between ISG15 expression and HCC cell migration (**, *P* < 0.01). The HCC cell migration was shown in the microscopic fields.

We developed ISG15 knock-down 97L cells (97L-shISG15) through transfection by pSUPER-shISG15 vector (Figure 3A, left panel) and ISG15 over-expression Huh7 cells (Huh7-ISG15) through transfection by pcDNA3.1-ISG15 vector (Figure 3A, right panel). Knocking down ISG15 markedly reduced incorporation of [^3^H]-thymidine into DNA of 97L cells at all time points compared with the control vector transfected cells (Figure 3B, left panel, *P* = 0.003). In contrast, ISG15 over-expression significantly increased incorporation of [^3^H]-thymidine into DNA of Huh7 cells (Figure 3B, right panel, *P* = 0.007). We then evaluated the effect of ISG15 on cell cycle using flow cytometry. The proportion of G2/M population in 97L-shISG15 cells was higher than that in control 97L cells (97L-shCtrl) (29.5% vs. 14.2% ), whereas Huh7-ISG15 cells in the G2/M population decreased from 27.3% to 13.1% compared to Huh7 cells (Huh7-Ctrl) (Figure 3C). Consequently, the cyclin B1 and cyclin dependent kinase-1 (CDK1) were also reduced after knocking down ISG15 in 97L cells (Figure 3D, left panel). Opposite results were obtained by using ISG15 over-expression Huh7 cells (Figure 3D, right panel).

As shown in Table 1, ISG15 overexpression significantly correlated with metastasis (*P* = 0.001). This result suggests that ISG15 is endowed with metastatic features. To test the hypothesis, we examined the migratory abilities of 97L-shISG15 and Huh7-ISG15 cells using transwell migration assay. 97L-shISG15 cells displayed approximately 3-fold lower cell migration efficiency than 97L-shCtrl cells (Figure 3E, upper panel, *P* < 0.01), and Huh7-ISG15 cells exhibited higher migration ability compared to Huh7-Ctrl cells (Figure 3E, lower panel, *P* < 0.01).

### ISG15 maintains Survivin protein stabilization via XIAP

**Figure 4:**
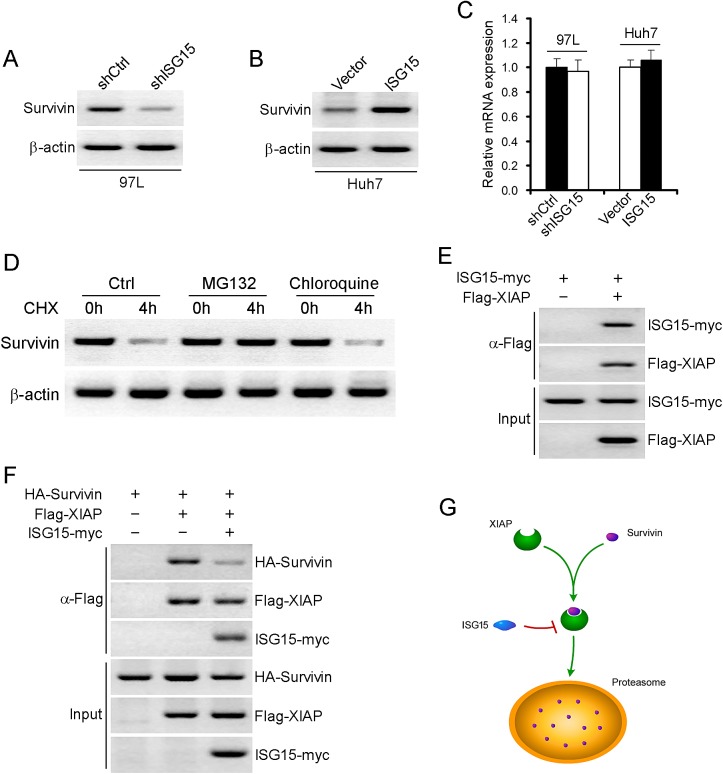
ISG15 maintains Survivin protein stabilization via XIAP Survivin protein was detected using western blotting in 97L-shISG15 cells (A) or Huh7-ISG15 cells (B). β-actin was probed as a negative control. (C) Survivin mRNA was detected using realtime-PCR in 97L-shISG15 cells and Huh7-ISG15 cells. β-actin was probed as a negative control. (*P* > 0.05). (D) Examination of Survivin protein degradation pathways. 97L cells were incubated with either a proteasome inhibitor (MG-132) or a lysosomal inhibitor (chloroquine). (E) Proteins from lysates were immunoprecipitated with antibody to Flag, and followed by immunoblotting with antibodies to myc. (F) Proteins from lysates were immunoprecipitated with antibody to Flag, and followed by immunoblotting with antibodies to HA, Flag and myc. (G) A model chart described the interaction between ISG15, Survivin and XIAP.

In our previous study, Survivin is highly expressed in HCC tissues [28]. We also found that the Survivin expression was significantly correlated with the expression of ISG15 (data not shown). To further explore the mechanism, we examined the protein levels of Survivin in 97L-shISG15 cells or Huh7-ISG15 cells. Survivin protein level was lower in 97L-shISG15 cells than that in 97L-shCtrl cell, and higher in Huh7-ISG15 cells than that in Huh7-Ctrl cells (Figure 4A, B). However, there were no difference in Survivin mRNA level between 97L-shISG15 and 97L-shCtrl cells or between Huh7-ISG15 and Huh7-Ctrl cells (Figure 4C, > 0.05), suggesting that Survivin was regulated at post-transcriptional level rather than at transcriptional level. To determine whether Survivin stability was affected by the proteasomal or lysosomal degradation pathway, 97L cells were incubated with either a proteasome inhibitor (MG-132) or a lysosomal inhibitor (chloroquine). MG132 but not chloroquine reversed Survivin protein levels, indicating that proteasomal degradation pathway was involved in the degradation of Survivin protein (Figure 4D). Moreover, we found direct interaction between recombinant ISG15 and XIAP (Figure 4E). We therefore propose that ISG15 may modulate Survivin ubiquitination via XIAP. Furthermore, we also found that ISG15 markedly weakened the association between XIAP and Survivin (Figure 4F), supporting the hypothesis that ISG15 sequesters XIAP from interacting with Survivin, thereby strengthens Survivin stability (Figure 4G).

### Knocking down ISG15 inhibits tumor growth, angiogenesis and extends tumor-bearing mice lifespan

**Figure 5:**
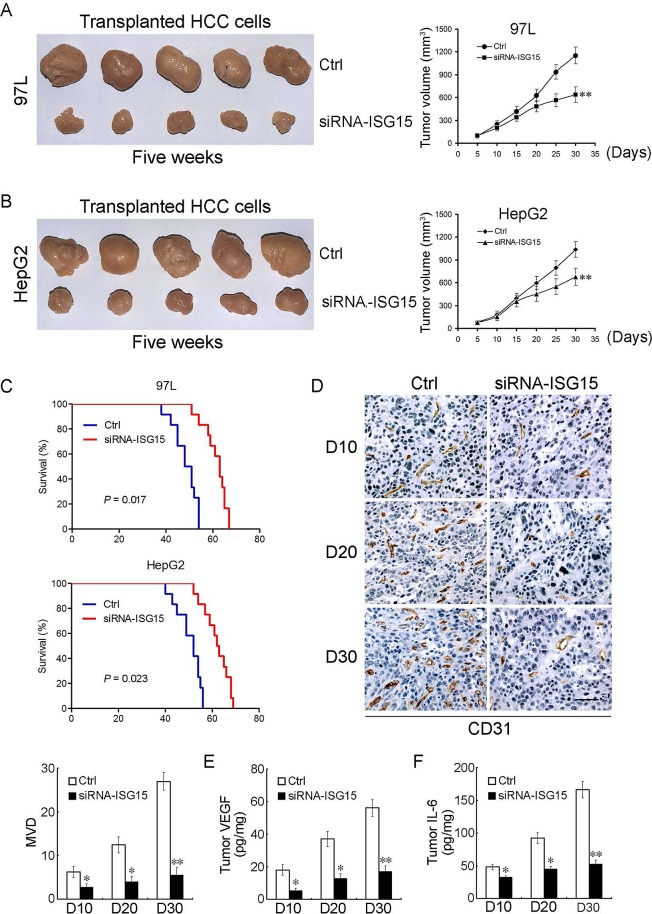
ISG15 silencing inhibits tumor growth, angiogenesis and prolongs tumor-bearing mice lifespan (A, B) Effect of ISG15 on HCC tumor growth in xenografted tumor models (**, *P* < 0.01). (C) Kaplan-Meier curves for overall survival were compared between siRNA-ISG15 and siRNA-Ctrl group (97L cells, *P* = 0.017 and HepG2 cells, *P* = 0.023, respectively, log-rank test, n = 12 per group). (D) The xenografted tumor tissues administered intratumor into siRNA-ISG15 or siRNA-Ctrl were stained by anti-CD31 antibody on days 10, 20, 30 for the quantification of mean MVD using immunohistochemistry. Data are the mean ± SD of mean MVD for siRNA-ISG15 or siRNA-Ctrl group as shown in the Histogram (n = 12 per group, *, *P* < 0.05; **, *P* < 0.01). (E, F) VEGF and IL-6 levels inside tumors were assayed by ELISA assay. VEGF and IL-6 levels inside tumors significantly decreased in siRNA-ISG15 treated mice (n = 12 per group, *, *P* < 0.05; **, *P* < 0.01).

To determine the *in vivo* effects of knocking down ISG15 on tumor growth, we established xenografted tumor models by subcutaneously injecting 97L or HepG2 cells into the back of BALB/c nude mice. When tumors reached a size of 0.3 to 0.5 cm in diameter, siRNA-ISG15 or siRNA-Ctrl was admistrated by intra-tumor injection. Silencing ISG15 significantly inhibited subcutaneous tumor growth compared with the control groups by injection of the empty vector. At the end of observation course (30 days), the inhibitory rate of cancerous growth for ISG15 knockdown was 55% and 65%, for 97L and HepG2 group, respectively (Figure 5A, B, *P* < 0.01). For survival analysis, siRNA-ISG15 can significantly prolong the survival rate of 97L or HepG2 tumor-bearing mice compared with the control group (Figure 5C, *P* < 0.05). To further investigate whether ISG15 affects the tumor microenvironment, we detected microvessel density (MVD) and cytokines such as vascular endothelial growth factor (VEGF) and Interleukin-6 (IL-6) in mouse models of HCC. siRNA-ISG15 significantly reduced mean MVD counts at the observed time points (Figure 5D). siRNA-ISG15 remarkably inhibited VEGF and IL-6 production within tumor tissues (Figure 5E, F). These data not only imply that siRNA-ISG15 could repress tumor proliferation and angiogenesis, but also from the reverse side further confirm that the elevated ISG15 could trigger metastasis of HCC.

## DISCUSSION

ISG15 is a type I interferon regulated gene that is induced by viral infection through the JAK/STAT signaling pathway [29, 30]. Previous studies have shown that ISG15 is associated with chemotactic activity towards neutrophils, direction of ligated target proteins to intermediate filaments, cell-to-cell signaling, and antiviral activity during viral infections [31, 32]. It was reported that ISG15 was highly expressed in multiple human cancer cell lines, and ISG15 plays crucial roles in modulating cell growth and progression of breast cancer [26, 33, 34]. However, the physiological or pathological functions of ISG15 in HCC have not been clearly elucidated.

Our findings suggested that ISG15 is involved in the proliferation and migration of HCC cells. These data cast light on novel mechanisms of HCC progression. ISG15 mRNA levels in HCC cells and tumor tissues from patients are higher than non-HCC cell and HCC adjacent tissues. Furthermore, using anti-ISG15 antibody clearly indicated ISG15 protein overexpression in HCC cells and tumor tissues from patients. Knocking down ISG15 by shRNA resulted in remarkable reduction of HCC cell proliferation. Concordantly, exogenous ISG15 expression in transfected cells promoted HCC cell growth. Moreover, we find that ISG15 is closely related to cell cycle. Cell cycle checkpoints are important control mechanisms that ensure the proper execution of cell cycle events. One of the checkpoints, the G2/M checkpoint blocks the entry into mitosis when DNA is damaged. Cyclin B1 and CDK1 are essential for G2/M phase transitions of eukaryotic cell cycle [35-37]. ISG15 knockdown can reduce the expression of Cyclin B1 and CDK1 and induce G2/M phase cell cycle arrest. In addition, ISG15 can also boost HCC cells migration. Survivin was originally identified as the smallest IAP family member. It can promote tumor cell proliferation, migration, invasion and counteract apoptosis *in vitro* and in transgenic animals [38, 39]. XIAP has been shown to function as an active center of E3 and to be responsible for the ubiquitination of substrates. A recent report demonstrated that Survivin and XIAP form a complex during cell death and synergistic inhibition of caspase activation [40, 41]. Consistent partially with this result, we found that the Survivin-XIAP complex existed in HCC cells. However, in our study, XIAP promoted the polyubiquitination of Survivin both *in vitro* and in intact cells (data not shown). We speculate that Survivin is one of the substrates of the E3 activity of XIAP. ISG15 interacts with XIAP, preventing XIAP and Survivin interaction, thereby keeping Survivin from proteasomal degradation pathway. This process ultimately results in HCC tumor progression.

We generated a siRNA against ISG15 and performed intratumor injection in tumor-bearing mice. This siRNA can not only inhibit tumor growth and but also prolong the survival time dramatically. Interestingly, ISG15 silencing affected tumor microenvironment including MVD and cytokines such as VEGF and IL-6. Previous studies have shown that MVD, VEGF and IL-6 are closely related to tumor proliferation and metastasis [42-44]. ISG15 silencing can also decrease MVD, VEGF and IL-6 *in vivo* obviously. These results show ISG15 was significantly correlated with the HCC proliferation and metastasis.

In summary, our results suggest that ISG15 pathway, aberrantly elevated in HCC, contributes to proliferation and metastasis of hepatocarcinoma cells via inhibiting targeted degradation of Survivin. ISG15 probably is a prognosis marker and its inhibition could develop a therapeutic advantage for HCC patients over-expressing ISG15.

## MATERIALS AND METHODS

### Patients and specimens

A total of 50 patients of HCC were recruited into this study with 44 males and 6 females. The mean age was 46.3 years (standard deviation, 9.6 years; range, 28 - 74 years). Tumor and para-cancerous tissues were procured from the potential curative tumor resection at the Hepatic Surgery Center of the Tongji Hospital Affiliated to Tongji Medical College, Huazhong University of Science and Technology in 2008. All patients were followed up for 5 year after surgery. All human studies were reviewed and approved by the Institutional Review Board at the Tongji Hospital and written informed consent was provided according to the World Medical Association Declaration of Helsinki. All the specimens were confirmed to be hepatocellular carcinoma by pathological examination. Tumor differentiation was graded by Edmondson-Steiner's criteria. Tumors with Edmondson-Steiner's grade I were considered as moderate to well differentiation and those with grade II-IV were poorly differentiated. Metastasis was defined as the involvement of extrahepatic tissue/organ and distant lymphadenopathy. Specimens were preserved in liquid nitrogen and formalin-fixed, paraffin-embedded blocks. Serial sections of 2-4 μm were prepared from the cut surface of blocks at the maximum cross-section of the tumor.

### Cells and animals

The HepG2 and Huh7 human hepatocellular carcinoma cell line and non-HCC L02 cell was purchased from the China Center for Type Culture Collection (CCTCC, Wuhan, China) and was cultured in 1640 complete culture medium (Gibco Inc., USA) with 10% newborn bovine serum (Invitrogen Inc., USA). MHCC97L Human hepatocellular carcinoma cell lines (97L) which have metastatic ability were purchased from Liver Cancer Institute, Zhongshan Hospital affiliated to Fudan University (Shanghai, china) [45]. The 97L cell lines were maintained as monolayer cultures in DMEM-high glucose medium supplemented with 10% fetal bovine serum (FBS), 100 IU/ml penicillin and 100 IU/ml streptomycin at 37°C in a humidified atmosphere, with 5% CO_2_. All cells were passaged for less than 6 months in our laboratory after receipt or resuscitation. BALB/c nude mice were obtained from the Animal Center of the Chinese Academy of Medical Science, Beijing.

### Immunoblot analysis and immunoprecipitation assay

For immunoblotting, immunoprecipitates or whole-cell lysates were resolved by Sodium salt-polyacrylamide gel electrophoresis (SDS-PAGE) and transferred to a polyvinylidene difluoride (PVDF) membrane (Bio-Rad). The immunoblots were probed with the following antibodies: anti-Flag (Sigma-Aldrich), anti-hemagglutinin (anti-HA) (Sigma-Aldrich), anti-myc (Sigma-Aldrich), anti-β-actin (Sigma-Aldrich) and anti-ISG15 (Santa Cruz). The proteins were visualized by using Western blotting system (Promega). For immunoprecipitation, cells were collected and then lysed in Nonidet P-40 buffer or sonicated in Tris-buffered saline (TBS) buffer supplemented with a complete protease inhibitor cocktail (Roche). After cell lysates were precleared with normal mouse IgG and protein A/G agarose beads for 1 h at 4°C, whole-cell lysates were used for immunoprecipitation with various antibodies. The primary antibodies were goat anti-human ISG15 (Santa Cruz Biotechnology, US), rabbit anti-human Cyclin B1 (Santa Cruz Biotechnology, US), rabbit anti-human CDK1 (Novocastra Laboratories Ltd, UK), mouse anti-human CD31 (Novocastra Laboratories Ltd, UK), and isotype-matched IgG (Sigma, Germany). Corresponding species-specific horseradish-peroxidase (HRP)-biotinylated (Pierce, US) were used. For immunoprecipitation assays, cells were transfected with the indicated plasmids. Plasmid-transfected cells were washed twice with phosphate-buffered saline and lysed in IP lysis buffer (20 mM Tris-HCl, pH 8.0, 150 mM NaCl, 1 mM EDTA, 0.5% Nonidet P-40, 1 mM dithiothreitol, and protease inhibitor mixture). Supernatants were immunoprecipitated with mouse IgG or anti-Flag antibody at 4°C overnight. The immunoprecipitated proteins were eluted from the protein A/G-agarose by boiling for 10 min in 1×SDS-PAGE sample buffer and immunoblotted with the indicated antibodies.

### Immunohistochemical staining

The tumor specimens were fixed in 10% buffered formalin and embedded in paraffin. Immunohistochemical staining was performed according to the manufacture's instruction. Briefly, the sections were deparaffinized in xylene and rehydrated in graded alcohol and distilled water. Subsequent to antigen retrieval, endogenous peroxidase activity was blocked with 0.3% hydrogen peroxide in methanol for 30 min, followed by rehydration in phosphate-buffered saline (PBS) and incubation with 5% goat serum for 60 min to bind the nonspecific antigens. The sections were incubated overnight at 4°C with the primary antibodies (anti-ISG15, 1/50, Santa Cruz). The immunosignals were detected using the ABC kit at room temperature. Subsequent to rinsing, the sections were incubated with 3,3-diaminobenzidine (DAB), counterstained with hematoxylin, dehydrated and mounted. The sections were then analyzed through standard light microscopy and taken photos. A scoring system was used to evaluate the immunoreactivity, as previously reported [46]. The percentage of positive cells was classified as follows: 0 for ≤5%, 1 for 6–25%, 2 for 26–50%, and 3 for ≥51%. The intensity of immunostaining was graded as follows: 0 for negative, 1 for weakly positive (light brown), 2 for moderately positive (brown), and 3 for strongly positive (dark brown). The overall immunostaining score was calculated as follows: immunoreactivity score (IRS) = percentage score × intensity score. The results were reported positive if the IRS is ≥3, and negative if the IRS is < 3. All slides were independently scored by pathologists who were blinded to the clinical data. A high level of concordance (90%) was achieved. In case of disagreement between the reviewers, the slides were reviewed by both pathologists untill a consensus view is achieved.

### Silencing and overexpressing of ISG15

For silencing of ISG15, the RNA sequence against ISG15 for RNAi were designed based on pSUPER system instructions (Oligoengine) and cloned into pSUPER-puro that expresses 19 nt hairpin-type short hairpin RNA (shRNA) with a 9 nt loop. ISG15 shRNA-encoding sequences were as follows: 5'-GATCCCCGCACCTA CGAGGTACGG CTTTCAAGAGAAGCCGTACC

TCGTAGGTGCTTTTTA-3' (ISG15, sense); 5'-AGCTTAAAAAGCACCTACGAGG

TACGGCTTCTCTTGAAAGCCGTA CCTCGTAGGTGCGGG-3' (ISG15, antisence). The inserted shRNAs (pSUPER-shISG15) were confirmed by DNA sequencing. The 97L cells were transfected by using Lipofectamin 2000 (Invitrogen, US) as described by the manufacturer. ISG15 silenced cells were selected with puromycin (Sigma, Germany). Empty vectors transfected cells were used as controls. For overexpression of ISG15, human ISG15 was cloned into pcDNA3.1 expression vector. The ISG15 expression vector or Ctrl vector was transfected into cells by Lipofectamine 2000 (Invitrogen) according to the manufacturer's instructions. The ISG15 mRNA and protein expressions were detected by PCR and western blot analysis, respectively.

### Cell proliferation assay

Proliferation of cells was measured using a [^3^H]-thymidine incorporation assay. The 97L-shCtrl and Huh7-Ctrl cells, 97L-shISG15 and Huh7-ISG15 cells (2×10^3^ cells/well) were seeded on 96-well culture plates, cultured until the cells reached 70% to 80% confluency, and then serum starved in DMEM for 24 h. After 72 h, cells were pulsed with [^3^H]-thymidine for 4 h. Cells were harvested and their [^3^H]-thymidine incorporation was measured in the liquid scintillation counter LKB1219.

### Cell migration assay

The cell motility assay was performed using Transwell inserts (6.5-mm diameter, 8-μm pore size polycarbonate membrane) obtained from Corning (Cambridge, MA, US). Cells (1×10^5^) in 0.5 ml serum-free medium were placed in the upper chamber, and the lower chamber was loaded with 0.8 ml medium containing 10% fetal bovine serum. Cells that migrated to the lower surface of filters were stained with Wright–Giemsa solution (Sigma-Aldrich), and five fields of each well were counted after 24h of incubation at 37°C with 5% CO_2_. Three wells were examined for each condition and cell type, and the experiments were repeated three times.

### Animal experiments

Female 6-week-old BALB/c nude mice with a body weight of approximately 15g were used and kept under specific pathogen-free conditions. Xenografts of 97L cells or HepG2 cells were produced by injecting tumor cells (1×10^6^ resuspended in PBS) subcutaneously into the back of the mice. When tumors reached a diameter of 3 to 5 mm, the mice transplanted with 97L cells or HepG2 cells were grouped (12 mice per group) and administered intratumor siRNA-ISG15 or siRNA-Ctrl three times per week. Tumor size was measured twice per week. The life span of tumor-bearing mice was recorded. At necropsy, the tumors were separated and were fixed with 10% buffered formalin and embedded with paraffin. The tumor was determined by examining serial sections of tumor tissue by microscopy. CD31 staining were counted as MVD, which was obtained by manually counting the positive foci for slides counterstained with hematoxylin. MVD was scored as the number of vessels found in the field, and the final score was the average of the three most vascularized areas on the high power field [47]. Total proteins are extracted from HCC tissues through tissue homogenate and removal of nonprotein components. VEGF or IL-6 ELISA kit (R&D Systems, Inc.) was used according to the manufacturer's instructions. The assay was performed in triplicate according to the manufacturer's recommended procedures.

### Statistical analysis

All values were presented as mean±SD. The relationship between the expression level of ISG15 and various clinicopathological factors was analyzed using the Mann-Whitney U test. The relationship between the expression of ISG15 and 5-year survival of HCC patients was analyzed by Pearson Chi-squared test. Kaplan-Meier analysis was used to estimate the cumulative cause-specific survival of tumor-bearing mice. The influence of ISG15 on the growth and migration of HCC was analyzed by the Student's *t* test. One-way analysis of variance (ANOVA) followed by Tukey's test or *t* test were used to determine the difference among treatments. All statistical analysis was performed using the SPSS 17.0 software package for Macintosh (SPSS Inc., Chicago, US). A *P* value less than 0.05 was considered statistically significant, and *P* value less than 0.01 was remarkably significant.

## Abbreviations List

DAB: 3,3-diaminobenzidine
Huh7-Ctrl: Control Huh7 cells
CDK1: Cyclin dependent kinase-1
FBS: Fetal bovine serum
HBV: Hepatitis B virus
HCC: Hepatocellular carcinoma
HRP: Horseradish-peroxidase
IRFs: IFN regulated factor
ISGs: IFN-stimulated genes
ISG15: IFN-stimulated genes 15
IFNs: Interferons
IL-6: Interleukin-6
97L-shISG15: ISG15 knock-down 97L cells
Huh7-ISG15: ISG15 over-expression Huh7 cells
97L: MHCC97L Human hepatocellular carcinoma cell lines
MVD: Microvascular density
PBS: Phosphatebuffered saline
PVDF: Polyvinylidene difluoride
PI: Propidium iodide
TBS: Sodium salt-polyacrylamide gel Tris-buffered saline
UCRP: Ubiquitin cross-reactive protein
VEGF: Vascular endothelial growth factor
XIAP: X-linked inhibitor of apoptosis
